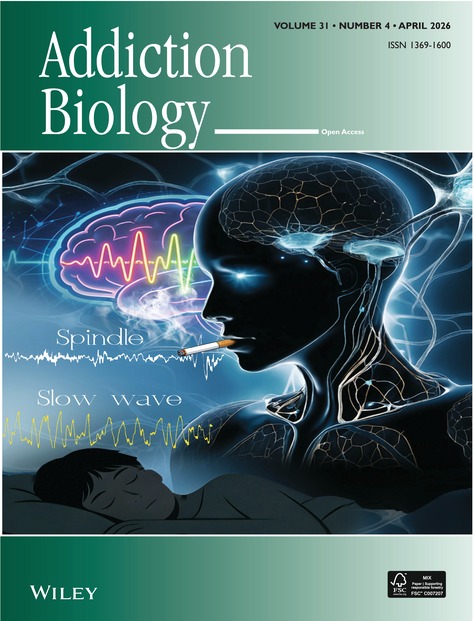# Cover Image

**DOI:** 10.1111/adb.70163

**Published:** 2026-04-30

## Abstract

The cover image is based on the article *Sleep Slow-Wave and Spindle Alterations in Young Smokers Correlated With the Severity of Cigarette Exposure* by Shuailin Ding et al., https://doi.org/10.1111/10.1111/adb.70149.